# Machine learning integration of single-cell and bulk transcriptomics identifies fibroblast-driven prognostic markers in colorectal cancer

**DOI:** 10.17305/bb.2025.12038

**Published:** 2025-04-22

**Authors:** Ning Zhang, Ruiyan Liu, Siya Wu, Chenxi Feng, Boxiang Wang, Qiaoqiao Zheng, Linru Jie, Ruihua Kang, Xiaoli Guo, Xiaoyang Wang, Shaokai Zhang, Jiangong Zhang

**Affiliations:** 1 Department of Cancer Epidemiology, The Affiliated Cancer Hospital of Zhengzhou University & Henan Cancer Hospital, Zhengzhou, China; 2 College of Public Health, Zhengzhou University, Zhengzhou, China

**Keywords:** Colorectal cancer, CRC, fibroblasts, prognosis signature, machine learning, therapy.

## Abstract

Single-cell RNA sequencing (scRNA-seq) has significantly advanced our understanding of cellular heterogeneity and the complex interplay within the tumor microenvironment (TME) of colorectal cancer (CRC). However, translating these molecular insights into clinically actionable prognostic biomarkers and therapeutic strategies remains a considerable challenge. In this study, we conducted a comprehensive scRNA-seq analysis of 306 CRC samples comprising 448,255 cells to characterize the TME in depth. By constructing intercellular communication networks based on connection counts and communication probabilities, we identified fibroblasts as central regulatory hubs within the TME. Using Wilcoxon rank-sum tests and univariate survival analyses, we initially identified 23 prognostic fibroblast markers. These were refined to a seven-gene fibroblast-related prognostic signature via an integrated machine learning approach. The signature exhibited robust predictive performance in the The Cancer Genome Atlas - Colon Adenocarcinoma (TCGA-COAD) training cohort (*n* ═ 351; C-index = 0.65) and was successfully validated in the GSE17536 dataset (*n* ═ 177; C-index = 0.63). Functional enrichment analyses revealed that this signature is involved in immune regulation and multiple tumor-associated cellular pathways. Notably, high-risk patients displayed increased macrophage and NK cell infiltration, impaired immune function, and elevated immune rejection scores, while low-risk patients demonstrated heightened sensitivity to camptothecin and irinotecan. Together, our findings underscore the prognostic value of fibroblast-derived signatures in CRC and support their potential utility in risk stratification and the development of personalized therapeutic strategies, contributing to the advancement of precision oncology.

## Introduction

According to GLOBOCAN 2022, colorectal cancer (CRC) ranks fourth in global cancer incidence and third in cancer-related mortality [1]. Despite advances in multimodal treatments [2, 3], patient outcomes remain poor—particularly for advanced cases, where the five-year survival rate is only 14% [4, 5]. This poor prognosis can be attributed to several key factors. Notably, tumor heterogeneity-induced differential treatment responses and acquired drug resistance have shifted research attention toward the tumor microenvironment (TME) as a critical determinant of both prognosis and therapeutic outcomes [6, 7]. Recent studies [8, 9] have shown that elements of the TME significantly influence CRC progression and treatment efficacy. Although various prediction models based on survival-associated genes have been developed, most focus exclusively on tumor cell characteristics [10], largely overlooking the TME’s crucial role. For instance, cancer-associated fibroblasts (CAFs) within the TME secrete growth factors and cytokines that promote tumor growth and metastasis [11]. Additionally, the composition of tumor-infiltrating lymphocytes has been closely associated with patient survival, with a higher density of CD8+ T cells typically indicating a better prognosis [12]. Conversely, regulatory T cells and myeloid-derived suppressor cells foster an immunosuppressive environment that hampers anti-tumor immune responses [13]. This immunosuppression is particularly relevant in the context of immunotherapy, as only about 15% of microsatellite instability-high CRC patients respond to immune checkpoint inhibitors (ICIs). Given these complex TME interactions, a deeper understanding and targeted analysis of key regulatory components are essential for improving prognostic assessments and optimizing personalized treatment strategies for CRC. Recent advances in single-cell RNA sequencing (scRNA-seq) [14] have significantly enhanced our ability to analyze TME complexity. By providing gene expression profiles at single-cell resolution [15], scRNA-seq enables detailed characterization of cellular subpopulations—as demonstrated in CRC research that identified distinct T cell exhaustion states [16]. Although bulk RNA sequencing [17] lacks single-cell resolution, it provides large-scale data that are vital for identifying clinical patterns. Therefore, integrating the high-resolution insights of scRNA-seq with the broad validation capabilities of bulk RNA-seq represents a powerful strategy for prognostic assessment in modern cancer research [18]. Accordingly, this study aims to integrate high-resolution scRNA-seq data with large-scale bulk RNA sequencing to gain a comprehensive understanding of TME complexity and identify key components within the CRC microenvironment. We will focus on the regulatory factors identified in our analyses and apply advanced machine learning techniques to improve prognostic risk stratification for CRC patients, offering new perspectives on potential therapeutic targets.

## Materials and methods

### scRNA-sequencing data collection and analysis

The scRNA-seq data used in this study were obtained from a comprehensive dataset integrating 15 independent CRC cohorts, compiled by Chu et al. [19]. This integrated dataset, comprising 671,192 cells and 51,971 genes, is publicly available on Figshare (https://figshare.com/). From this dataset, we extracted all 204 tumor core tissue samples and 102 adjacent non-cancerous tissue samples. Sample types were classified based on the metadata provided in the original dataset. Batch effects had already been addressed and corrected using the Harmony algorithm, ensuring sample homogeneity across different cohorts. Quality control excluded cells with fewer than 1000 detected genes, mitochondrial gene content exceeding 20%, or red blood cell gene content above 3%. scRNA-seq analysis was performed using Seurat v4.0 [20] on the tumor core tissue samples. The RunUMAP function was used for nonlinear dimensionality reduction and visualization of the gene expression matrix. Cell clustering was conducted using the Louvain algorithm with a resolution parameter of 0.2. Cell annotation was based on both the original dataset metadata and differential gene expression analysis using the FindAllMarkers function. Subsequently, we applied CellChat analysis to the tumor core tissue samples to characterize intercellular communication networks within the CRC TME. Leveraging curated ligand-receptor interaction databases, we quantified communication probabilities across various signaling pathways. A comparative analysis between the 204 tumor core samples and the 102 adjacent non-cancerous samples was performed to identify key cellular components involved in TME interactions.

### Bulk RNA-seq data collection

Bulk RNA-seq datasets The Cancer Genome Atlas-Colon Adenocarcinoma (TCGA-COAD) [21] and GSE17536 [22] were obtained from TCGA and GEO, respectively. The training dataset included 351 CRC patient samples from TCGA-COAD, selected based on survival duration greater than one month and the availability of complete gene expression data. An external validation cohort comprising 177 samples from GSE17536 was identified using the same inclusion criteria.

### Machine learning-driven integrative signature development

Following CellChat analysis that highlighted the critical role of fibroblasts in the TME, we investigated their prognostic value in CRC patients. Using the Wilcoxon rank-sum test, we identified fibroblast-specific marker (FSM) genes based on stringent criteria (logFC ≥ 2, min.pct ≥ 0.25, FDR < 0.05). These FSM genes were then subjected to univariate Cox regression analysis using the TCGA-COAD dataset to pinpoint potential prognostic markers. To construct a robust risk scoring system, we applied an integrated machine learning approach incorporating 10 algorithms: Random Survival Forest, Elastic Net, Lasso regression, Ridge regression, Stepwise Cox regression, CoxBoost, Partial Least Squares Regression Cox model, Supervised Principal Component Analysis, Gradient Boosting Machine, and Survival-SVM. This integration involved evaluating 101 algorithm combinations through leave-one-out cross-validation (LOOCV). Risk scores were calculated as linear combinations of gene expression levels, and model performance was assessed using the Concordance index (C-index). Top-performing combinations were selected based on their validation set C-index and potential for clinical translation, resulting in the development of a fibroblast-related signature (FRS) for predicting overall survival riskin CRC patients. To validate the FRS as an independent prognostic factor, we compared its ROC curves with those of other clinical characteristics. Additionally, we developed an integrated nomogram combining the FRS with clinical features to estimate survival outcomes in CRC patients.

### Enrichment analysis

To explore the potential functions of FRS and its associated biological pathways, we used STRING [23] to predict genes that may interact with the FRS gene, defining these as FRSR genes. We then conducted functional enrichment analysis using KEGG pathways and GO terms to investigate the biological roles these genes may play in tumor development. In this study, KEGG pathway and GO enrichment analyses were performed using the OmicShare platform, an integrated online tool that offers comprehensive bioinformatics analysis with user-friendly visualization features.

### Exploration of immune characteristics

To investigate the correlation between FRS and immune cell infiltration in the CRC TME, we quantified the infiltration levels of 22 immune cell types using the CIBERSORT algorithm [24]. To validate the reliability and accuracy of the CIBERSORT results, we conducted cross-validation using five additional algorithms: EPIC [25], ESTIMATE [26], MCP-counter [27], quanTIseq [27], and TIMER [28]. To evaluate immunogenicity based on immunomodulators, immunosuppressive cells, and effector cells, we assessed the immune response profile using TIDE score computation, which predicts patient responses to immunotherapy based on integrated gene expression data. TIDE scores for TCGA-COAD patient samples were obtained from the TIDE database (http://tide.dfci.harvard.edu/).

### Drug discovery and sensitivity analysis

To identify candidate therapeutic agents, we utilized the Drug Signature Database (DSigDB) [29] to screen for compounds targeting FRS-associated genes. We then applied the oncoPredict package to assess chemotherapeutic sensitivity in CRC patients stratified by FRS risk scores. This approach allowed us to estimate drug-specific IC50 values based on gene expression profiles, thereby supporting individualized predictions of drug response.

### Statistical analysis

R software (version 4.4.0, R Foundation for Statistical Computing, Vienna, Austria) was used for the primary analytical procedures, including data manipulation, statistical computations, and visualization. The analysis workflow incorporated a comprehensive suite of specialized R packages: Seurat for processing scRNA-seq data and correcting batch effects; dplyr, stringr, tidyverse, and reshape2 for data manipulation; scRNAtoolVis, ggplot2, ggpubr, and ComplexHeatmap for data visualization; DoubletFinder for doublet detection; CellChat for intercellular communication analysis; limma and Mime1 for machine learning model integration and construction; oncoPredict for drug sensitivity prediction; IOBR for immune infiltration analysis; survival, survminer, and ggDCA for survival analysis; and org.Hs.eg.db and msigdbr for annotation and pathway analysis. Enrichment analyses were conducted using the OmicShare platform. For statistical analysis, normality was first assessed using the Shapiro–Wilk test, which indicated non-normal data distribution (*P* < 0.05). As a result, the Wilcoxon rank-sum test was applied for paired group comparisons, with data presented as median ± interquartile range (IQR). Nomograms were constructed using multivariate Cox regression analysis, and enrichment analysis employed hypergeometric testing to identify pathways or terms significantly enriched among differentially expressed genes compared to the genome-wide background. All statistical tests were two-sided, with α < 0.05 considered statistically significant.

## Results

### T cells and epithelial cells constitute the primary cellular components of CRC tissues

**Figure 1.**
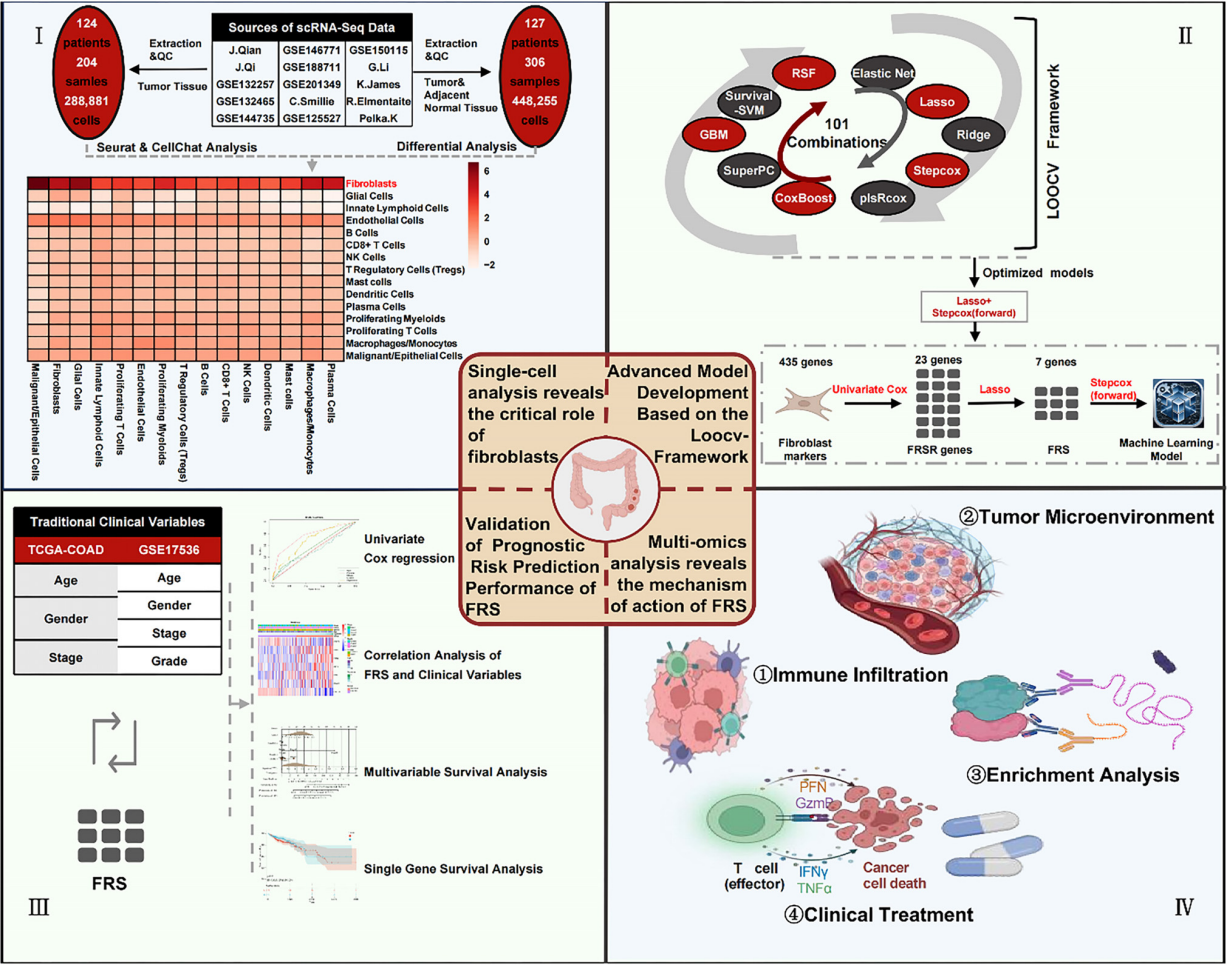
**Abstract figure of this study.** Integrated analysis of colorectal cancer tumor microenvironment and development of key cellular signatures for overall survival prediction and personalized therapy. QC refers to quality control, FRSR genes refers to FRS-related genes. Abbreviations: FRS: Fibroblast-related signature; TCGA-COAD: The Cancer Genome Atlas-Colon Adenocarcinoma; scRNA-seq: Single-cell RNA sequencing.

**Figure 2.**
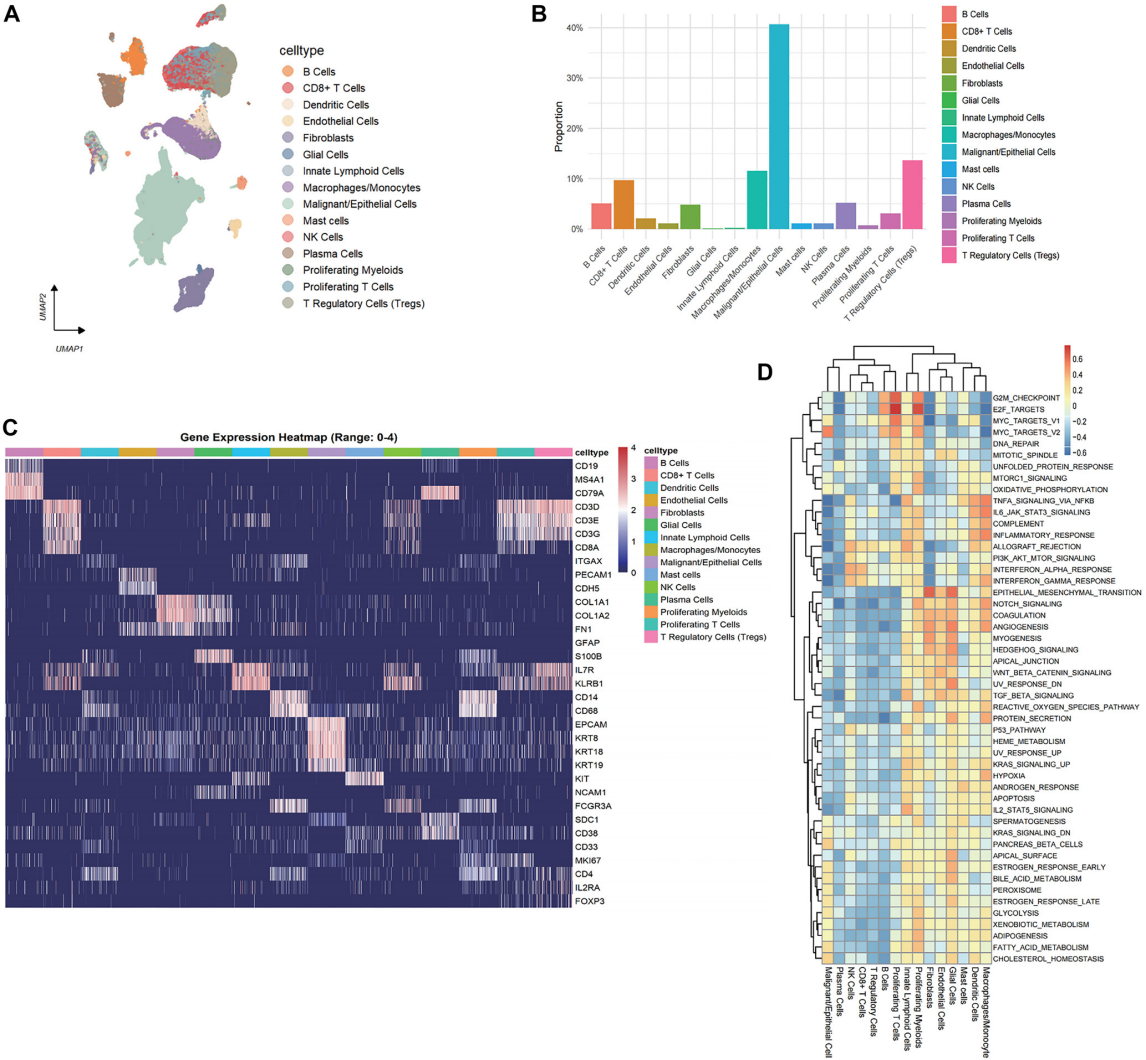
**Overview of various cell types in the TME of CRC at the scRNA transcriptome level.** (A) Identification of 15 cell types in 204 CRC tumor center tissues; (B) Bar plot showing the distribution proportion of each cell type; (C) Heatmap showing the expression distribution of classic marker genes for each cell type; (D) ssGSEA score from hallmark gene sets of Human MSigDB Collections, changes in the heatmap cell colors indicate the level of activity of the corresponding pathways in specific cell types. Abbreviations: CRC: Colorectal cancer; TME: Tumor microenvironment.

A graphical abstract of this study is presented in Figure 1. To comprehensively characterize the TME of CRC and delineate its cellular heterogeneity, we conducted an integrated analysis of scRNA-seq data from 204 tumor core samples across 15 independent datasets. Using the UMAP algorithm, we identified 21 distinct cell clusters (Figures S1A–S1C). Further analysis revealed 15 unique cell types (Figure 2A), with T cells and malignant/epithelial cells predominating in the tumor tissue. Notable immune populations included monocytes/macrophages (11.6%) and NK cells (11.0%) (Figure 2B). To validate the accuracy of cell type annotations, we generated a heatmap of key marker gene expression across the identified cell types (Figure 2C). Additionally, Gene Set Variation Analysis (GSVA) showed that proliferating myeloid and T cells were significantly enriched for cell cycle-associated gene sets. Notably, fibroblasts exhibited strong upregulation of EMT signatures, suggesting a potential role in promoting tumor progression and metastasis (Figure 2D).

### Cellchat analysis highlights fibroblasts as key regulators in CRC TME

**Figure 3.**
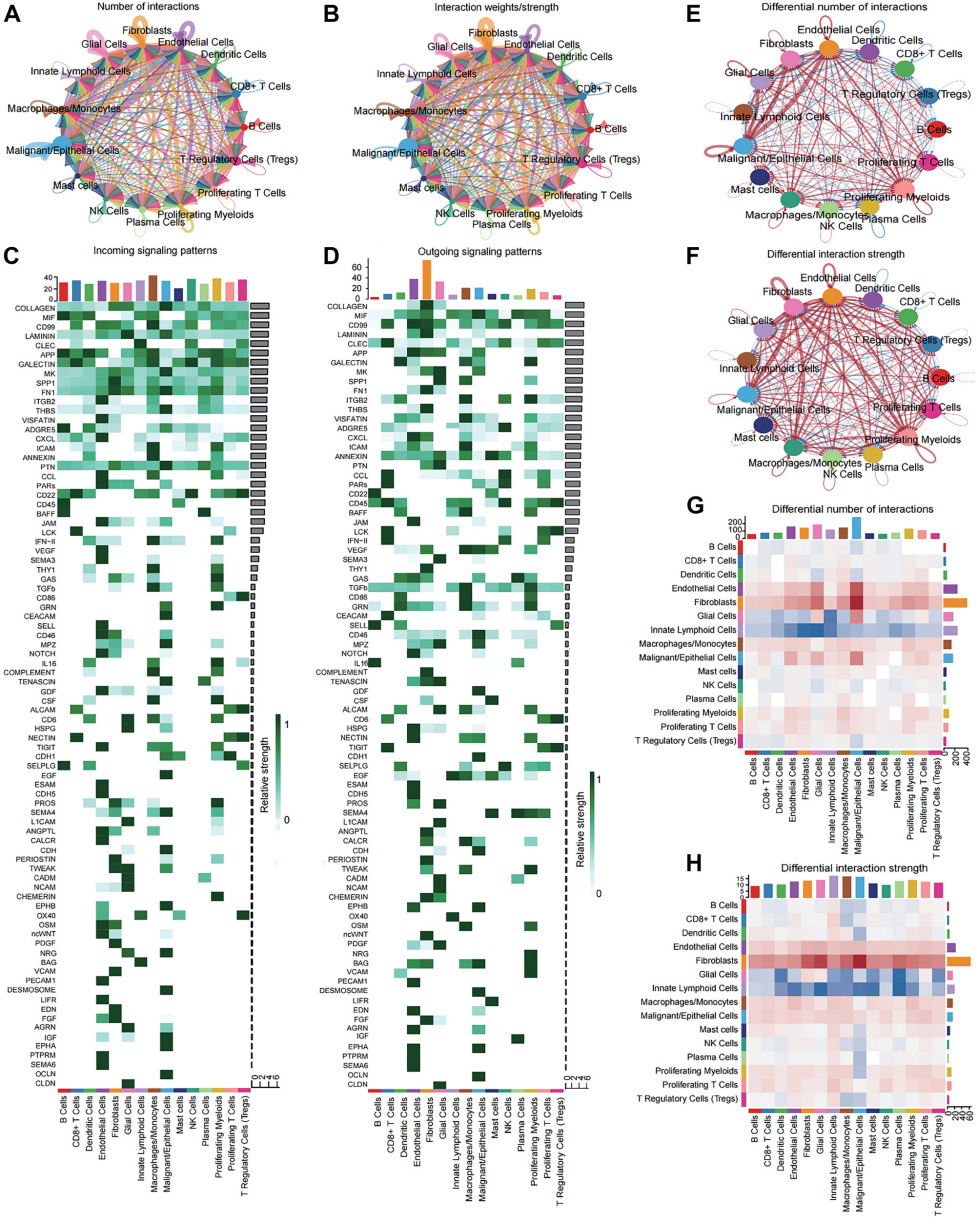
**CellChat reveals the crucial role of fibroblasts in the TME of CRC.** The circular network diagram displays the net number (A) and strength (B) of intercellular communication among different cell types in tumor tissue. Colors represent different cell types, while the thickness of the lines indicates the magnitude of change. Heatmaps detail the contribution of signaling molecules, separately showing incoming (C) and outgoing (D) signals for each cell type. Circular plots display the differential networks of net number (E) and interaction strengths (F) of cell-cell interactions between tumor core and adjacent normal tissues. Blue indicates decrease, red indicates increase, and the thickness of the lines represents the magnitude of change. The heatmap provides a more intuitive representation of the variations in the quantity (G) and intensity (H) of interactions between tumor core and adjacent normal tissues. Abbreviations: CRC: Colorectal cancer; TME: Tumor microenvironment.

To identify the essential regulatory components within the CRC TME, we performed CellChat analysis on 204 tumor core tissues to systematically investigate patterns of intercellular communication. Our analysis revealed that, in CRC tumor tissues, fibroblasts and endothelial cells exhibited higher net interaction counts and interaction weights compared to other cell types (Figure 3A and 3B). These findings suggest that these two cell types function as central regulators, modulating the activity and behavior of other cells within the TME. Further analysis showed that fibroblasts contributed the most to outgoing signals in the cellular communication network (Figure S2A and S2B), underscoring their dominant role. We also observed significant variations in both incoming and outgoing signal contributions across different cell groups (Figure 3C and 3D). To further elucidate key differences in cellular communication between tumor and normal tissues, we compared the 204 tumor core tissues with 102 adjacent normal tissues. We found that, in tumor tissues, the net interaction counts and weights among fibroblasts, endothelial cells, and malignant/epithelial cells were significantly increased (Figure 3E and 3F). Notably, changes in fibroblast activity were especially pronounced (Figure 3G and 3H), indicating that fibroblasts may play a critical regulatory role in CRC tumor progression by enhancing their interactions with other key cell types.

### A robust seven-gene FRS predicts CRC prognosis using the LOOCV framework

**Figure 4.**
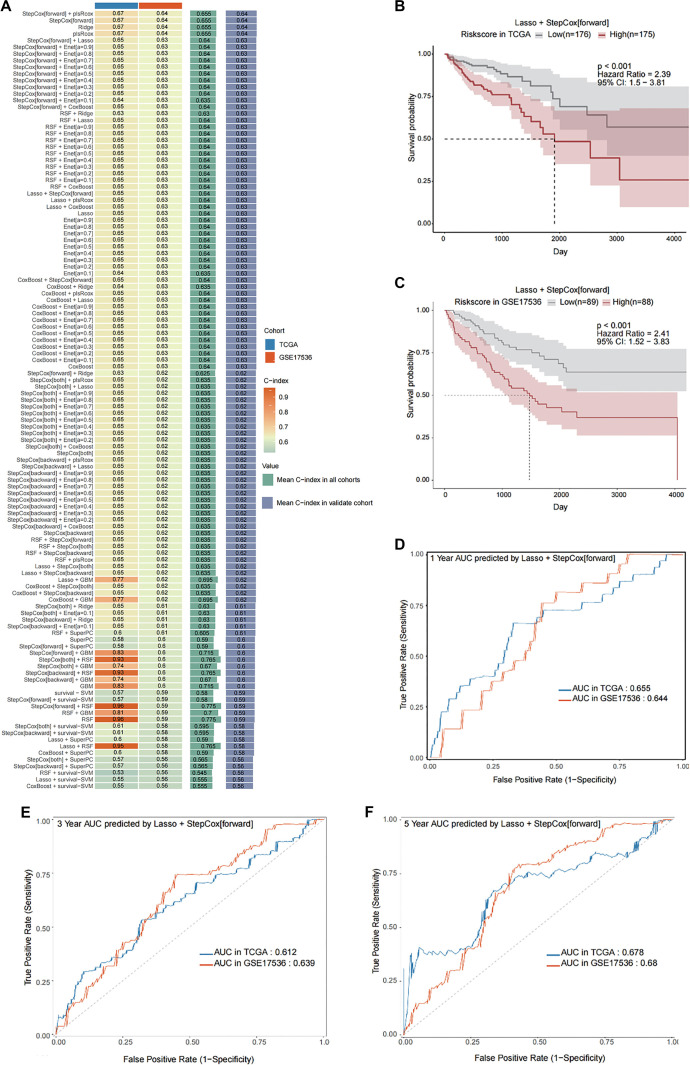
**Establishment and validation of a consensus FRS through a machine learning-based integrated pipeline.** (A) Total of 101 types of prediction models using the LOOCV framework and further calculated the C-index of each model in all validation datasets. Kaplan–Meier survival curves for different risk groups in TCGA-COAD (B) and GSE17536 (C). The ROC curves illustrate the OS performance at one year (D), three years (E), and five years (F) in the two datasets. Abbreviations: LOOCV: Leave-one-out cross-validation; C-index: Concordance index; TCGA-COAD: The Cancer Genome Atlas-Colon Adenocarcinoma.

Acknowledging the central role of fibroblasts in cellular interactions, we explored the potential value of the FRS in predicting the prognosis of CRC patients. Using the FindMarkers function, we identified 435 FSM genes that are highly expressed in fibroblasts within tumor tissue. We then performed univariate Cox regression analysis on these FSM genes in the TCGA-COAD cohort, which yielded 23 potential prognostic biomarkers. Subsequently, these markers were incorporated into 101 combination models using an LOOCV framework. The predictive performance of each model was evaluated by calculating the C-index in both the training and validation sets (Figure 4A). All models were ranked based on their C-indices in the validation set. While four combination models incorporating all 23 biomarkers demonstrated optimal predictive performance (C-index ═ 0.64; Figure 4A), we sought to develop a more clinically applicable signature. To enhance translational potential and minimize overfitting caused by multiple correlated genes, we conducted a comprehensive model selection process. This analysis revealed that a more parsimonious model—combining Lasso regression with forward stepwise Cox regression and including only seven genes—achieved comparable predictive performance (C-index ═ 0.63). Based on these findings, we selected the Lasso+StepCox[forward] model as the optimal approach and developed a seven-gene FRS for predicting prognosis in CRC patients. The FRS derived from the Lasso+StepCox model stratified patients into high- and low-risk groups using median scores. Survival analyses demonstrated significantly poorer outcomes in high-risk patients across both training and validation sets (HR ═ 2.39 and 2.41, respectively; both *P* < 0.001; Figure 4B and 4C). The robustness of the FRS as a prognostic tool was further supported by time-dependent ROC analysis, which demonstrated consistent predictive accuracy for progression-free survival at one-year (AUC in TCGA ═ 0.655, AUC in GSE17536 ═ 0.644), three-year (AUC in TCGA ═ 0.612, AUC in GSE17536 ═ 0.639), and five-year (AUC in TCGA ═ 0.678, AUC in GSE17536 ═ 0.68) intervals (Figure 4D–4F). Expression validation of the seven FRS genes in fibroblasts across independent scRNA-seq datasets is shown in Figure S4A–S4I.

### Integration of FRS with clinical characteristics improves prognostic accuracy in CRC

**Figure 5.**
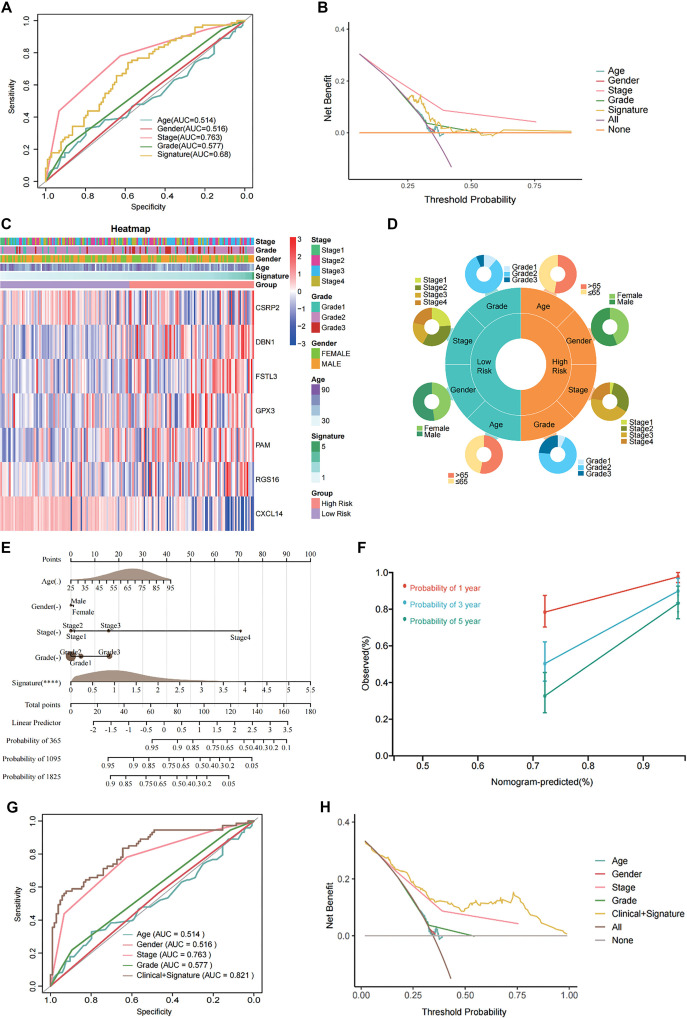
**Compared to traditional clinical variables, FRS demonstrates potential as an independent prognostic factor in GSE17536.** Univariate ROC curve (A) and DCA curve (B) of clinical characteristics and signature; (C) The distribution of clinical characteristics and the expression of model genes according to the FRS risk score; (D) Correlation between the FRS low- and high-risk groups and clinical characteristics; (E) Construction of the nomogram based on the FRS and clinical characteristics, including age, gender, grade and stage; (F) Calibration curve of the nomogram for one-, three-, and five-year OS. ROC curve (G) and DCA curve (H) shows the prediction performance between the nomogram and Clinical characteristics. Abbreviations: DCA: Decision curve analysis; FRS: Fibroblast-related signature.

To evaluate the prognostic value of FRS relative to conventional clinical characteristics, we compared its performance with factors, such as age, gender, tumor grade, and stage in the GSE17536 dataset. Univariate Cox regression analysis indicated that FRS had superior predictive accuracy (AUC ═ 0.68) compared to these clinical features, as reflected by higher AUC values (Figure 5A). Decision curve analysis (DCA) further demonstrated that FRS provided greater net clinical benefit across low-risk thresholds (threshold < 0.5, Figure 5B). Stratification analysis also revealed significant differences in tumor stage and grade distribution between FRS-defined risk subgroups (Figure 5C and 5D). To improve the clinical utility of FRS, we constructed a nomogram combining FRS with clinical variables using Cox regression analysis (Figure 5E). This model achieved a C-index of 0.81 (95% CI: 0.76–0.85), with FRS remaining an independent prognostic factor in multivariate analysis (*P* < 0.001). Calibration curves indicated that the nomogram had better predictive accuracy for one-year survival compared to three- and five-year survival (Figure 5F). These results were independently validated in the TCGA-COAD dataset (Figure S3A–S3F). To further explore the individual prognostic contributions of FRS-related genes, we performed univariate Cox regression analysis on the seven signature genes. Elevated expression of CSRP2, DBN1, FSTL3, GPX3, PAM, and RGS16 was associated with poorer prognosis, whereas CXCL14 showed a protective effect (Figure S5A). Among them, CSRP2 was the strongest predictor (HR ═ 1.94, 95% CI: 1.34–2.82). Clustering based on risk scores revealed consistent patterns across gene expression profiles, risk stratification, and survival outcomes (Figure S5B and S5C).

### FRS-related genes are enriched in immune regulation and cellular signaling pathways

**Figure 6.**
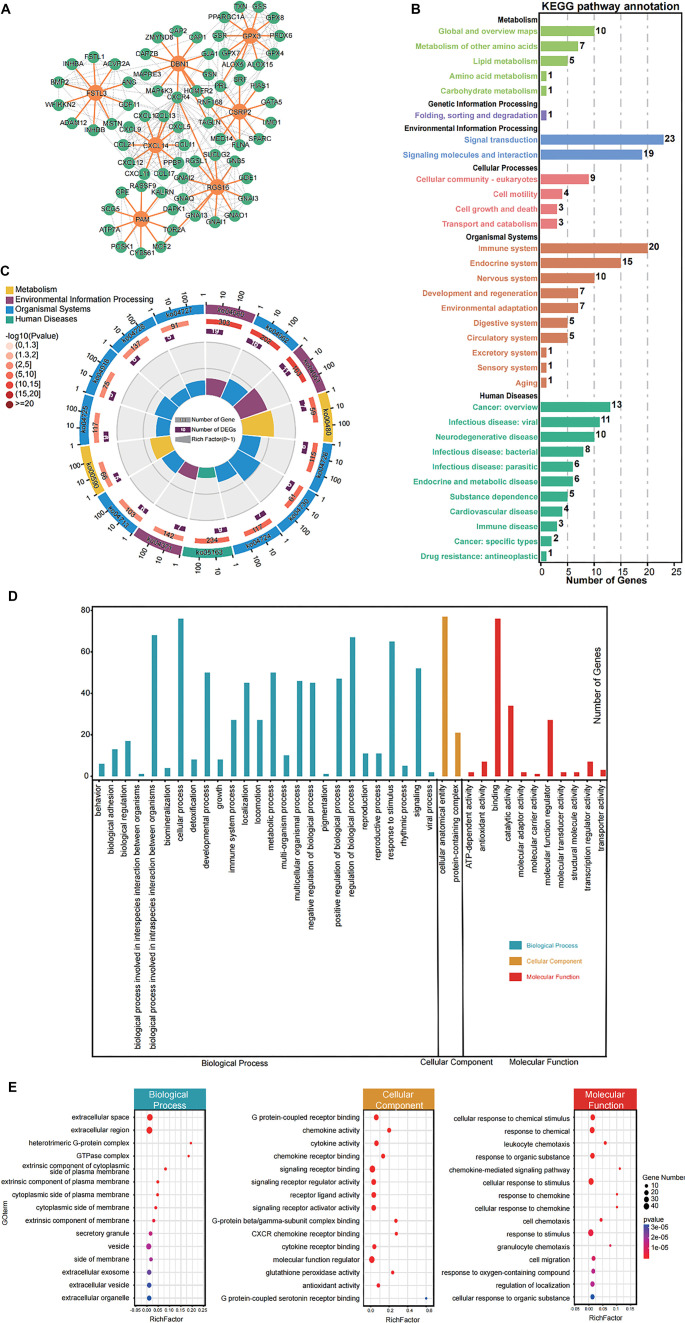
**Functional enrichment analysis reveals the potential molecular mechanisms by which FRS affects prognosis.** (A) PPI network of FRSR genes predicted through String database; (B) The number of DEGs in various pathways in KEGG analysis; (C) KEGG enrichment circle diagram of cyan module (from the outside to the inside, the first circle represents the top 15 enrichment pathways, and the number outside the circle is the coordinate ruler of the number of genes; The second circle represents the number and *Q* value of background genes in this pathway, and the more genes, the longer the bar; The third circle represents the number of the DEGs in this pathway; The fourth circle represents the value of rich factor in each pathway); (D) The number of DEGs in various terms in GO enrichment analysis; (E) Enrichment of the top 15 gene sets (ranked by *P* value) across the three primary categories of GO enrichment analysis. Abbreviations: DEG: Differentially expressed gene; FRS: Fibroblast-related signature.

To elucidate the fundamental mechanisms by which FRS influences the prognosis of CRC patients, we analyzed genes interacting with FRS (designated as FRSR genes) using the STRING database (Figure 6A). KEGG enrichment analysis of each module revealed that the primary categories were signal transduction, the immune system, and signaling molecules and interaction (Figure 6B). Further analysis indicated that the most significantly enriched pathways for FRSR genes were Cytokine–cytokine receptor interaction (ko04060) and Chemokine signaling pathway (ko04062) (Figure 6C), suggesting that FRSR genes may regulate cancer-related processes by modulating immune system functions. Similarly, GO enrichment analysis was conducted on FRSR genes, categorizing terms with *P* < 0.05 into three main aspects: biological processes, cellular components, and molecular functions (Figure 6D). The results showed that FRSR genes were predominantly enriched in cellular process (GO:0009987), cellular anatomical entity (GO:0110165), and binding (GO:0005488). Moreover, bubble plot visualization demonstrated that FRSR genes are primarily involved in cellular signal transduction and immune response, emphasizing their broad roles in fundamental cellular activities (Figure 6E). Collectively, these findings, along with the KEGG pathway analysis, further support the hypothesis that FRSR genes may influence tumor progression through immune regulation.

### FRS correlates with immune microenvironment characteristics and predicts immunotherapy efficacy

**Figure 7.**
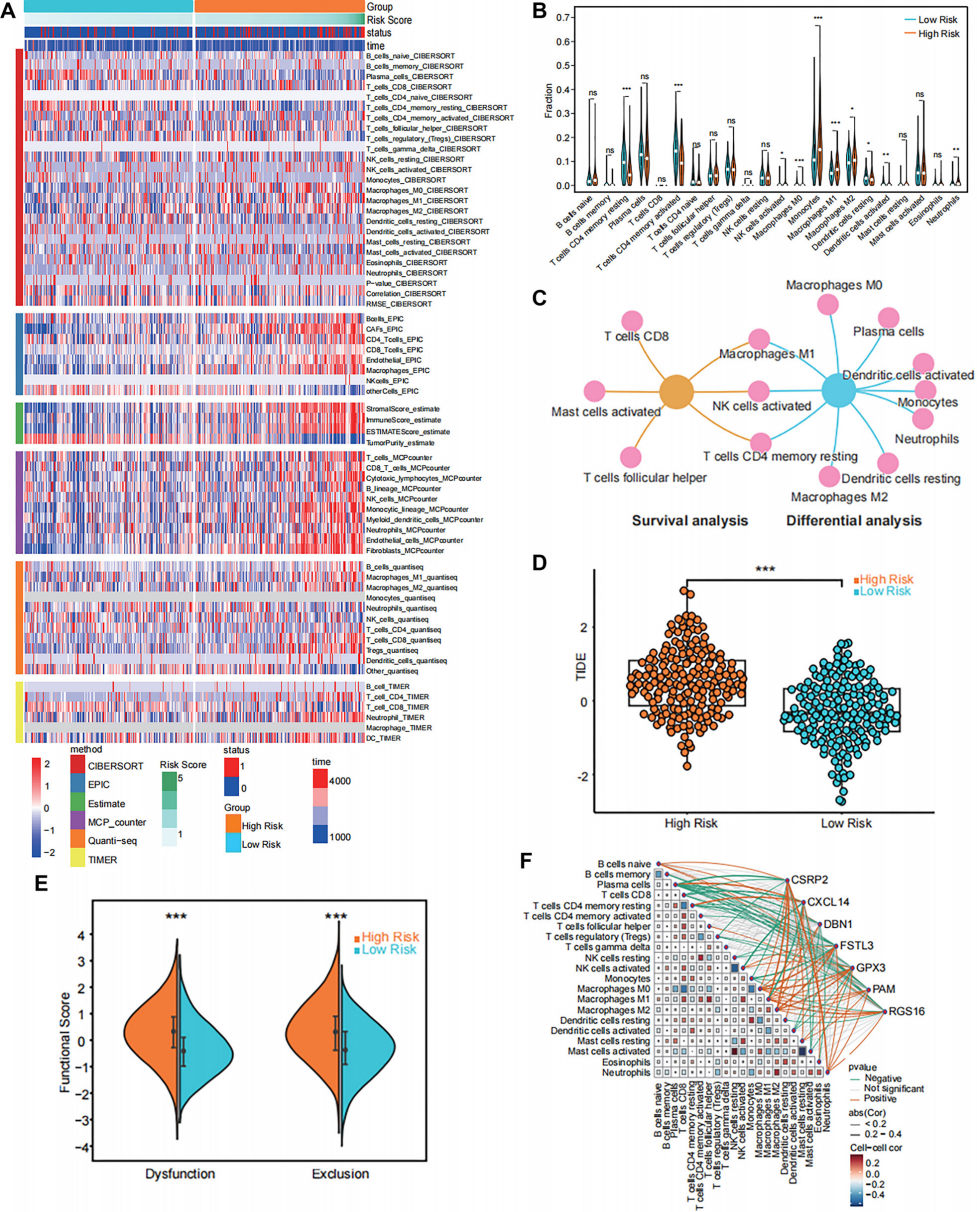
**The immune landscape associated with FRS in CRC.** (A) The heatmap shows the immune infiltration landscape of six methods under different FRS risk subgroups; (B) The abundance of each type of tumor-infiltrating immune cell in high-risk and low-risk groups, ns (not significant), *(*P* < 0.05), **(*P* < 0.01), ***(*P* < 0.001); (C) Types of infiltrating cells in the TME intersecting with differential analysis and survival analysis; (D) TIDE scores for each sample within different risk subgroups, TIDE refers to the TIDE score derived from the TIDE database. ns (not significant), *(*P* < 0.05), **(*P* < 0.01), ***(*P* < 0.001); (E) T-cell dysfunction and exclusion scores between high- and low-risk groups, ns (not significant), *(*P* < 0.05), **(*P* < 0.01), ***(*P* < 0.001); (F) The network diagram of the correlation between immune cell subsets and gene expression. (The correlation between specific cell and FRS genes is represented by different colored lines, with orange indicating positivity and green indicating negativity. The thickness of the lines reflects the strength of the correlation. Abs [52] represents the absolute value of the correlation coefficient, and Cell-cell cor indicates the correlation coefficient between different cell types.) Abbreviations: FRS: Fibroblast-related signature; CRC: Colorectal cancer.

To evaluate the impact of the FRS on immune cell infiltration in CRC patients, we employed the CIBERSORT algorithm to quantify immune cell abundance in TCGA-COAD samples. Cross-validation with five additional algorithms confirmed the reliability of the results (Figure 7A), demonstrating consistency across different analytical methods. Notably, under the ESTIMATE algorithm, the high-risk group exhibited significantly higher stromal score, immune score, and estimate score, but lower tumor purity compared to the low-risk group (Figure 7A). Wilcoxon rank-sum test analysis revealed statistically significant differences in the infiltration of 10 immune cell types between the risk subgroups (Figure 7B). In particular, the high-risk group showed increased infiltration of activated NK cells and macrophage subsets, suggesting that differences in immune cell infiltration may influence CRC prognosis. We further analyzed the association between immune cell infiltration and overall survival in CRC patients. The results identified six immune cell types significantly associated with patient prognosis (Figure S6B–S6G). By integrating differential infiltration data (Figure 7B) with survival analysis (Figure S6B–S6G), we identified three key immune microenvironment cell types: M1 macrophages, activated NK cells, and resting memory CD4+ T cells (Figure 7C). To explore the potential relationship between the prognostic biomarkers and immunotherapy response, we applied the TIDE algorithm to predict immune escape scores in the training set. The analysis revealed that patients in the high-risk group had significantly higher immune escape scores (*P* < 0.001) (Figure 7D), suggesting that these tumors may be more capable of evading immune surveillance. Further evaluation of two key TIDE indicators showed that both T cell dysfunction and T cell rejection scores were significantly elevated in the high-risk group (*P* < 0.001) (Figure 7E). These findings indicate that high-risk patients may exhibit a poorer response to immunotherapy, aligning with their worse survival outcomes. Additionally, to further understand the interplay between FRS genes and the immune microenvironment, we constructed a network diagram illustrating correlations between these genes and various immune cell subsets (Figure 7F). Notably, genes, such as FSTL3 and CSRP2 displayed strong positive correlations with multiple immune cell subsets, particularly macrophage subsets and neutrophils. These findings suggest that certain genes may play a pivotal role in modulating immune responses in CRC.

### FRS-targeted therapeutic agents reveal distinct drug response patterns in different risk groups

**Figure 8.**
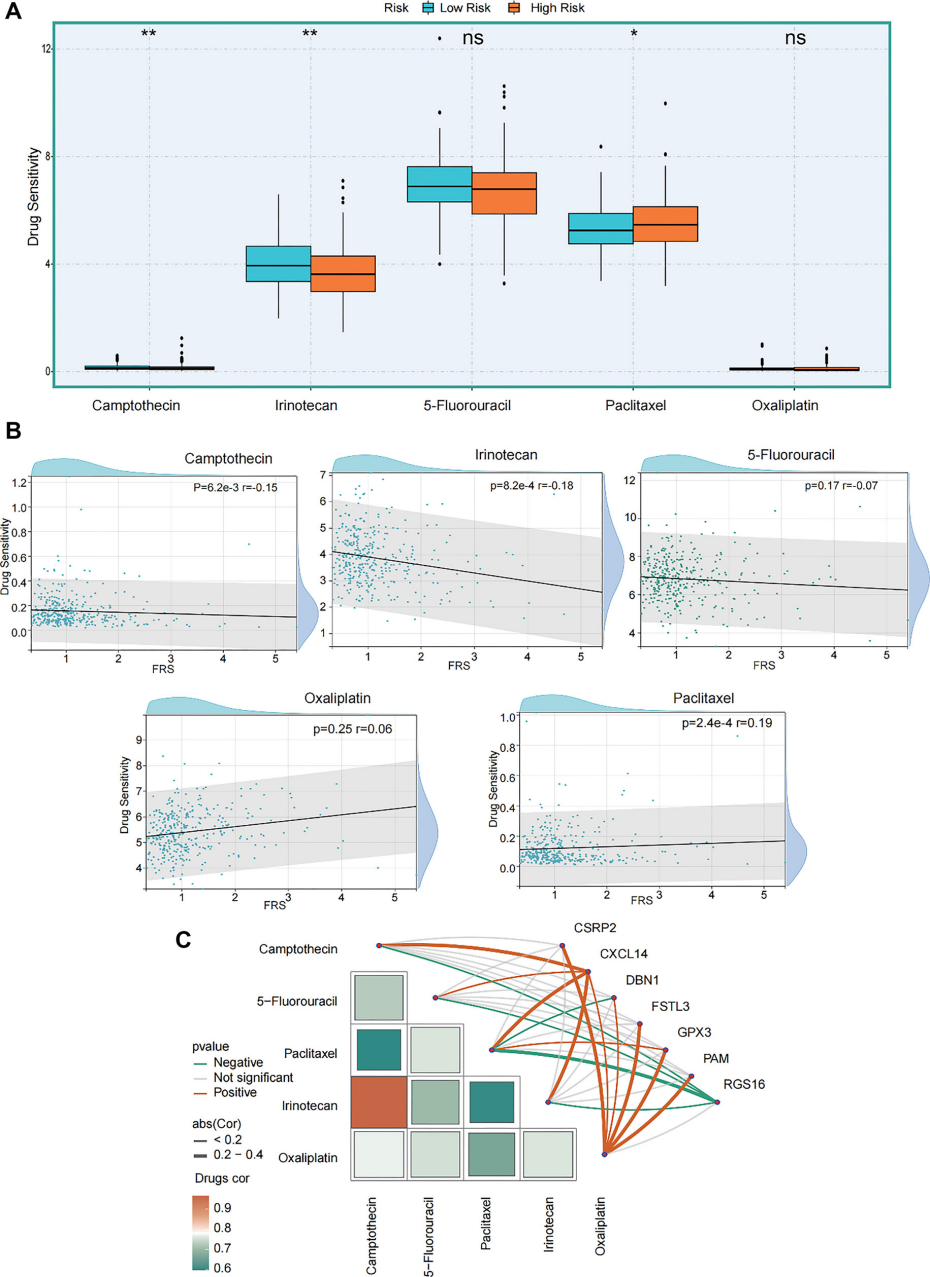
**The drug sensitivity associated with FRS in CRC.** (A) Drug sensitivity of five compounds calculated by the oncoPredict function across different FRS subgroups, ns (not significant), *(*P* < 0.05), **(*P* < 0.01), ***(*P* < 0.001); (B) The scatter plot shows the sensitivity of the samples to five compounds as a function of FRS; (C) The network heatmap reflects the relationship between drug sensitivity and gene expression. (Abs represents the absolute value of the correlation coefficient, and Drugs cor represents the correlation coefficient between different drugs.) Abbreviations: FRS: Fibroblast-related signature; CRC: Colorectal cancer.

To identify potential therapeutic agents targeting FRS, we analyzed the DSigDB database and ranked candidate drugs based on *P* values. The ten most significant candidates are listed in Table 1. Among them, four anticancer agents that are widely used in clinical practice were identified: camptothecin, irinotecan, sanguinarine, and daunorubicin. Notably, camptothecin and irinotecan—both topoisomerase I inhibitors—are commonly employed in the clinical treatment of CRC. Given that the dynamic and heterogeneous nature of the TME can contribute to drug resistance, we evaluated the sensitivity of FRS-defined risk subgroups to five commonly used chemotherapeutic agents for CRC: camptothecin, irinotecan, and three standard treatments—5-fluorouracil, paclitaxel, and oxaliplatin (Figure 8A). The results revealed that the low-risk group exhibited significantly greater sensitivity to camptothecin and irinotecan (*P* < 0.01). In contrast, the high-risk group showed increased sensitivity to paclitaxel (*P* < 0.05). A scatter plot analysis (Figure 8B) of FRS risk scores vs drug sensitivity produced consistent findings: as the risk score increased, tumor cell sensitivity to camptothecin (*P* < 0.001) and irinotecan (*P* <0.001) significantly declined, while sensitivity to paclitaxel increased markedly (*P* < 0.001). Furthermore, detailed correlation analyses demonstrated a significant positive association between oxaliplatin sensitivity and the expression patterns of several FRS genes (Figure 8C), suggesting that these genes may play distinct roles in modulating tumor cell responses to different chemotherapeutic agents.

**Table 1:** Drug prediction based on intrinsic target genes of FRS from DSigDB

**Term**	***P* value**	**Odds ratio^a^**	**Combined score^b^**	**Genes^c^**
Estradiol	<0.001	109,648	1,173,816	CSRP2; GPX3; RGS16; CXCL14; PAM; DBN1; FSTL3
Camptothecin	<0.001	67.7	676.8	GPX3; RGS16; FSTL3
Ellipticine	<0.001	100.8	806.6	CSRP2; RGS16
15-delta prostaglandin	<0.001	66.2	475.8	CSRP2; GPX3
Irinotecan	0.0012	21.7	146.1	GPX3; RGS16; FSTL3
Sanguinarine	0.0019	40.8	254.9	CSRP2; RGS16
Progesterone	0.0023	12.6	76.6	CSRP2; GPX3; CXCL14; DBN1
Daunorubicin	0.0027	34.4	203.2	CSRP2; RGS16
3-nitrobenzanthrone	0.0038	333.1	1852.2	CXCL14
Arecoline hydrobromide	0.0042	302.8	1657.4	RGS16

^*a^Odds Ratio: A measure of the association strength between the term and target genes. ^b^Combined Score: A comprehensive indicator, higher scores indicate more reliable associations between the term and target genes. ^c^Genes: Genes that interact with the drug. Abbreviations: FRS: Fibroblast-related signature; DSigDB: Drug Signature Database.

## Discussion

In this study, we analyzed integrated scRNA-seq data and applied methods, such as ssGSEA and CellChat to investigate the heterogeneity of the TME in CRC. Our systematic single-cell analysis revealed the functional diversity of fibroblasts within the TME and enabled the identification of specific marker genes. Building upon these findings, we developed a novel prognostic signature composed of seven fibroblast-related genes by integrating multiple machine learning algorithms. Notably, this FRS demonstrated strong prognostic performance in both the training set and independent validation cohorts, outperforming traditional clinical variables and showing robustness across algorithmic frameworks, including Lasso + CoxBoost and Lasso + plsRcox. Beyond its predictive power, our study underscores the critical role of FRS in CRC prognosis and provides insight into its potential mechanisms, particularly its modulation of the tumor immune microenvironment. These findings offer a theoretical foundation for developing personalized treatment strategies based on FRS expression patterns. Fibroblasts, abundant within the TME, play key roles in CRC initiation and progression [30]. Consistent with our enrichment analyses, prior studies [31, 32] have shown that fibroblasts promote a tumor-favorable environment through angiogenesis, immune modulation, and matrix remodeling—underscoring their prognostic relevance in CRC. Moreover, fibroblast-derived extracellular matrix (ECM) proteins and matrix-remodeling enzymes such as MMPs not only create physical barriers but also increase matrix stiffness and interstitial pressure [33], thereby hindering the efficacy of chemotherapeutic and targeted agents. The accumulation of aberrant ECM components further intensifies immunosuppression and diminishes the effectiveness of ICIs [34], highlighting the importance of stratifying patients based on FRSs and developing targeted therapies for fibroblast-defined subgroups. Unlike the study by Zhang et al. [35], which used bulk RNA-seq and WGCNA to develop a 20-gene CRC fibroblast-related prognostic signature (with validation cohort AUCs of 0.638 and 0.55), our approach employed scRNA-seq data and an LOOCV framework that integrated 10 machine learning algorithms and their combinations. This approach reduced variable complexity and led to the identification of a consensus seven-gene FRS with improved predictive performance. The signature achieved a three-year AUC of 0.639 and demonstrated strong clinical translational potential. Integration of the FRS with clinical variables in a nomogram yielded notable improvements in net benefit, supporting its promise as a precision medicine tool for CRC survival prediction. Our findings also revealed substantial differences between FRS-defined subgroups in terms of immune response and cancer progression. The high-risk group exhibited elevated infiltration of activated NK cells and specific macrophage subsets, suggesting compensatory activation of innate immunity in the context of impaired adaptive immune responses. However, this compensatory mechanism appears insufficient to curb tumor progression and may instead facilitate TME formation via chronic inflammation [36]. Reduced infiltration of plasma cells and resting memory CD4+ T cells in this group indicated compromised adaptive immunity, potentially affecting tumor-specific antibody production, immune memory formation, and responsiveness to immune CIs [37]. High-risk patients showed greater immune evasion during checkpoint blockade therapy, characterized by diminished T cell infiltration and functional impairments in infiltrating T cells. This supports the existence of a dual immunosuppressive mechanism in the high-risk TME: both limited T cell infiltration and dysfunction of the T cells that do infiltrate.

Notably, all genes included in our constructed FRS have been previously reported to be closely associated with immune responses or tumor development. DBN1 is significantly overexpressed in the CRCE1 cell line, and immunohistochemical experiments have validated its association with CRC metastasis [38]. FSTL3, which contains a follistatin-like domain [39], promotes tumor invasion and metastasis by modulating key EMT molecules via the TGF-β1 signaling pathway. Studies have shown that FSTL3 is significantly elevated in CRC tissues, particularly in high-grade tumors [40, 41]. Although GPX3 negatively correlates with cholesterol levels, it is significantly elevated in poorly differentiated and advanced CRC patients, influencing CRC progression by regulating the cholesterol–T cell immune axis [42]. PAM, a bifunctional enzyme commonly dysregulated in cancer [43], was investigated by Zhang et al. [44], who identified three distinct PAM expression patterns, each associated with unique prognostic outcomes and TME characteristics across 1224 CRC samples. Notably, the high PAM subgroup correlates with advanced tumor stage, immunosuppressive cell infiltration, and poor prognosis. RGS16, characterized by a conserved RH domain and α-helix structure [45], shows elevated expression linked to poor overall survival in CRC patients [46]. Research indicates that RGS16 inhibits JNK/P38-mediated apoptosis in CRC cells by disrupting TAB2/TAK1 recruitment to TRAF6 [47]. CSRP2, a member of the CSRP protein family, is expressed at lower levels in CRC tissues compared to adjacent non-tumor tissues. Functional experiments have confirmed its inhibitory effects on CRC cell proliferation, migration, and invasion [48]. Interestingly, our findings suggest a tumor-promoting role for CSRP2, which contrasts with previous reports—potentially due to tumor heterogeneity and multifactorial influences. CXCL14, a key chemokine family member, plays an essential role in immune regulation and the TME [49]. In CRC, CXCL14 is primarily downregulated via epigenetic silencing and exerts tumor-suppressive effects by inhibiting EMT and regulating cell cycle progression. Low CXCL14 expression has been linked to poor prognosis in CRC patients [50]. Another key finding from our computational analysis is the identification of camptothecin and its semisynthetic derivative irinotecan as potential targeted therapeutics for FRS in CRC. This aligns with current clinical practice, where irinotecan-based FOLFIRI [51] and oxaliplatin-based FOLFOX [52] regimens are standard treatments. Our analysis suggests enhanced efficacy of camptothecin and irinotecan in CRC patients with low FRS scores, supporting a molecular classification-based approach to treatment selection. The therapeutic benefit of these agents is strongly supported by a multicenter randomized controlled trial by Chai et al. [53], which demonstrated that in metastatic CRC patients who failed 5-FU treatment, irinotecan monotherapy significantly prolonged median overall survival (9.2 months vs 6.5 months), and improved both objective response rate (13% vs 0%) and disease control rate (49% vs 21%) compared to best supportive care. However, TME heterogeneity frequently contributes to treatment resistance, compromising therapeutic efficacy. Therefore, these findings warrant comprehensive clinical validation—particularly through prospective clinical trials stratifying patients by FRS risk score—to directly compare sensitivity to camptothecin and irinotecan across molecular subtypes. Such studies would provide definitive evidence for implementing this molecular classification-based approach in personalized CRC treatment.

Our investigation highlights the pivotal role of FRS in guiding targeted prevention and personalized medicine for CRC. The findings suggest that FRS can provide valuable insights to support clinicians in making individualized treatment decisions, potentially improving patient outcomes while reducing unnecessary healthcare costs. However, this study has several limitations. First, although the FRS was evaluated and validated in both training and external cohorts, further confirmation through large-scale, multicenter prospective studies is necessary. Second, additional *in vitro* and *in vivo* research will be essential to clarify the biological mechanisms underlying FRS-related genes in CRC. Third, while we assessed the sensitivity of different FRS risk subgroups to various small-molecule drugs, these predictions require validation through *in vitro* drug testing and clinical trials. Fourth, although fibroblasts were identified as key regulators in the TME, our single-cell analysis did not explore the heterogeneity within fibroblast populations. A more detailed characterization of fibroblast subtypes—including their unique molecular profiles and functional states—could offer deeper insights into their diverse roles in tumor progression and enhance the prognostic utility of our signature. Future research should aim to develop a more nuanced classification of stromal cell populations to further refine risk stratification strategies. Collectively, these limitations outline key directions for future investigation.

## Conclusion

This study presents a comprehensive scRNA-seq analysis of CRC, highlighting the pivotal role of fibroblasts in the TME. By developing a novel seven-gene FRS, we introduce a robust prognostic tool that accurately predicts patient survival and informs potential personalized treatment strategies. The FRS signature underscores the complex interactions among fibroblasts, immune cells, and cancer progression, offering new avenues for precision medicine in the management of CRC.

## Supplemental data

Supplemental data are available at the following link: https://www.bjbms.org/ojs/index.php/bjbms/article/view/12038/3846.

## Data Availability

The data acquisition methods for this study have been mentioned in the text, and researchers can obtain additional data and analysis scripts by contacting the corresponding author.
